# Optimizing ex situ genetic resource collections for European livestock conservation

**DOI:** 10.1111/jbg.12368

**Published:** 2018-12-11

**Authors:** Rafael De Oliveira Silva, Bouda Vosough Ahmadi, Sipke Joost Hiemstra, Dominic Moran

**Affiliations:** ^1^ Global Academy of Agriculture and Food Security The University of Edinburgh Midlothian, Edinburgh UK; ^2^ Land Economy, Environment and Society Research Group Scotland’s Rural College (SRUC) Edinburgh UK; ^3^ Centre for Genetic Resources Wageningen University & Research Wageningen The Netherlands

**Keywords:** cryoconservation, ex situ conservation, gene bank, livestock diversity, optimization

## Abstract

Ex situ collections offer the potential to reduce extinction risks, affording option to society in maintaining future breeding opportunities for productivity and heritage traits. However, how much should we be seeking to collect and conserve in gene banks, and where? We developed a mathematical model to optimize logistical decisions of breed conservation choices and to evaluate alternative scenarios for efficiently re‐allocating genetic materials currently stored in different European gene banks, allowing for cross‐country collections, cost and cryogenic capacity differentials. We show how alternative allocations for the breeds that are currently stored in 11 European gene banks could reduce overall conservation costs by around 20% by selecting cryogenic banks that have relatively lower combination of fixed and collection costs, and are geographically closer to collection regions. Our results show that centralizing collections in one gene bank would double the costs, relative to collective European collections approaches. We also calculate marginal costs of collections and show that increasing diversity within the gene banks implies in higher costs per conserved breed.

## 1 INTRODUCTION

The increasing vulnerability of in situ animal and plant genetic resources for agriculture has been suggested by many authors, emphasizing the threats posed by climate change and increasing homogenization of farming and food production systems (FAO, 2015). The resilience of animal and plant varieties used for agriculture can potentially be maintained by ex situ collection of genetic and reproductive materials that can be used to improve and potentially to restore breeds. Gene banks complement in situ conservation, and include formal and informal use and exchange of genomic (e.g., DNA, blood, tissue) and reproductive germplasm (e.g., semen, embryos). Globally there are many agricultural biobank collections, typically held for specialized productive purposes. Some national and regional specialized collections emphasize indigenous and cultural breed attributes, for example, Rare Breeds Survival Trust (http://www.rbst.org.uk) in the UK. Other national and global plant and animal collections are held as public good resources in networks under The International Treaty on Plant Genetic Resources for Food and Agriculture (FAO, 2009), or the centres of Consultative Group on International Agricultural Research (http://www.cgiar.org). But other national and regional collections are more proprietary, offering restricted access usually through subscription. More generally, the academic literature on ex situ conservation is skewed towards storage of plant materials. There have been limited efforts to detail or audit animal collections. The European Gene bank Network for Animal Genetic Resources (EUGENA), coordinated by the European Regional Focal Point on Animal Genetic Resources (http://www.rfp-europe.org), is an emerging networking activity specifically targeting national farm animal genetic resource collections (Hiemstra, Martyniuk, Duchev, & Begemann, 2014). Furthermore, a recent survey conducted as part of the EU IMAGE (http://www.imageh2020.eu/) collaborative project (Passemard, Joly, Duclos, & Danchin‐Burge, 2018) elicited responses from 62 European organizations in 21 countries. The survey revealed some 30 genomic and 51 germplasm collections, with 20% of the organizations holding both germplasm and genomic materials. The data showed over‐ representation of some countries (e.g., Spain with 26 germplasm and 7 genomic collections).

Beyond Europe, The United States Department of Agriculture supports a National Animal Germplasm Program storing genetic material for use by industry and the research community (Blackburn, 2009). However, collection does not appear to be guided by any clear economic criteria beyond a budget constraint. FAO (2015) collects global data on stored genetic materials of various breeds, but depends on voluntary country reports that are often incomplete. Groeneveld et al. (2016) reviewed bio banking effort for all species and note a lack of a unified and generalised approach to sample collections in the domesticated animal sector.

There is general agreement that ex situ collections offer option value, that is, value of preserving a back‐up collection of (threatened) breeds so that this genetic diversity might be available for use in the future. But the efficacy of collections is also largely anecdotal, with some concern that materials stored in gene banks may be compromised, or become redundant or mismatched with independently evolved in situ conditions (McGowan, Traylor‐Holzer, & Leus, 2017). A further caveat on option value is the extent of overlap and possible redundancy in collections covering countries of similar agri‐climatic systems. In a collaborative system of material exchange this redundancy increases the cost of supplying diversity (Blackburn, 2009). While there is considerable focus on the efficiency of in situ biodiversity conservation, including area selection algorithms for systematic conservation planning (Kukkala & Moilanen, 2013; Önal, 2003) or conservation funds allocation (Reist‐Marti, Abdulai, & Simianer, 2006) we are unaware of work optimizing ex situ livestock collections. Specifically, the ex situ literature is apparently limited to optimizing genetic variability; that is, which breeds to conserve. However, as noted by Blackburn (2009) the logistical dimension of collections is an important but neglected limiting factor.

This paper develops a Mixed Integer Programming (MIP), a type of optimization model consisting of both integer (stored breeds) and continuous variables (e.g., costs) as opposed to Linear Programing (LP) which contains only continuous variables (Lee & Letchford, 2007). We use the model to identify the least cost collection and storage strategies for European livestock breeds under a collective budget constraint, and allowing cross‐country collections. We use the analysis to construct diversity supply curves to illustrate the relationship between cost and diversity in hypothetical rationalised ex situ collections. The analysis goes beyond existing ex situ cost exercises that have not considered the efficiency of potentially rationalised collections in a collaborative network (Pizzi, Turri, Gliozzi, & Gandini, 2016). The paper is structured as follows. Section two sets out the theoretical model for rationalization of collection effort, the survey data used in the analysis, and develops some scenarios for breed collection. Sections three and four provide results and discussion respectively, while section five offers conclusions.

## 2 MATERIAL AND METHODS

### 2.1 A model of optimized collections

Ex situ conservation decisions may be driven by several criteria including location‐specific biodiversity conservation targets, species or breed endangerment status, and economic and socio‐cultural weights (i.e., which breeds are more valuable for productive and other physiognomic traits). Logistical considerations are also inevitable and include conservation budgets or collection and maintenance costs of gene banks (GBs). The management decision may be further complicated by rationalization options; that is, keeping fewer collections at one or several geographical locations. This objective might be reasonable in the context of a collaborative research network where the free exchange and use of materials are a common goal. This study the has two objectives: (a) to identify least‐cost logistical strategies for material collection and storage; and (b) to estimate diversity costs for different livestock species, measured as the cost per conserved breed. Mathematical modelling can be used to rationalize multiple genetic collections covering overlapping material at several locations.

### 2.2 Model overview

To represent ex situ diversity and logistical decisions in MIP analysis we use two objective functions, namely cost minimization and diversity maximization. The latter is defined as the number of breeds that are collected and stored across a set of GBs. This is akin to a typical *facility location problem* (Geoffrion & Graves, 1974), which consists of selecting distribution centres along with their associated customer zones. The objective is to select facility sites to minimize distribution costs of demanded products. These costs typically include a part that is proportional to the sum of the distances from customer zones to the servicing facilities, plus costs of opening facilities at the chosen sites. The facilities may or may not have limited service capacities, which in turn distinguishes the problems in terms capacitated and uncapacitated variants. This problem is usually modelled as NP‐hard, meaning it is computationally difficult to solve, typically requiring specific algorithms (Shen, Zhan, & Zhang, 2011). In this application we formulate the analogous problem as a simple MIP, where the facilities and customer zones are respectively analogous to the gene banks and farm zones containing the breeds demanded to be conserved in gene banks.

**Figure 1:**
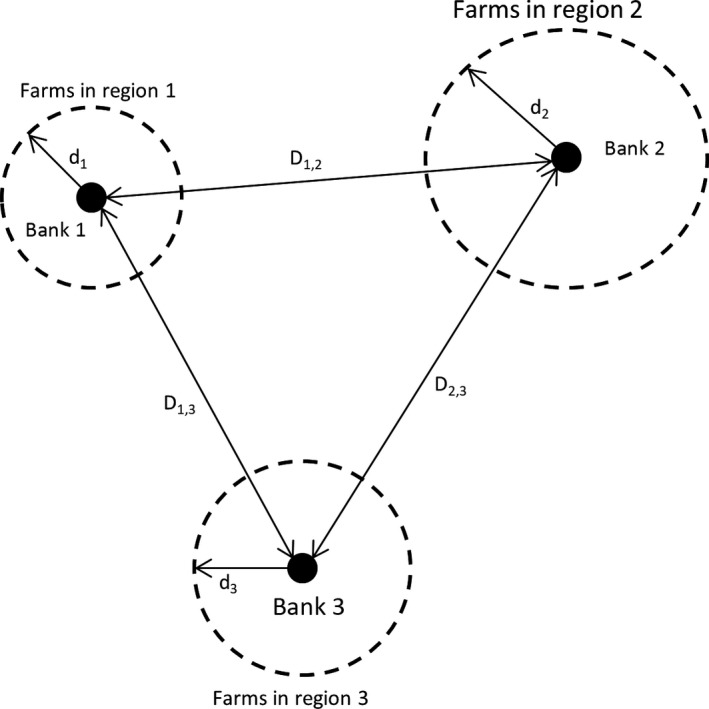
Conceptualization of gene bank optimization considering three illustrative gene banks (BANK 1–3), distance between the banks (e.g., *D*_1,2_) and distance from gene banks to farm zones, for example, *d*_1_

Figure 1 summarizes the MIP conceptualization and sets out the logistical decision process for selecting breeds for ex situ conservation.

Let BANKS 1–3 represent the GBs involved in a collective conservation program. BANK 1, located in region 1 is constrained to collect breeds that are in region 1 covering an average distance to farm locations, d_1_. Alternatively, BANK 1 can collect breeds at collection points, in this case d_1_ = 0; that is, breeds are brought to the bank for genetic material collection depending on travel and associated logistical costs. BANK 1 can also collect genetic materials from breeds in region 2 or region 3 by travelling 2D_1,2_ + 2d_2_ or 2D_1,3_ + 2d_3_, respectively. Collection decisions for BANK 2, BANK 3, and generalization to *n* banks are analogous to the BANK 1 case. The collection and storage of genetic materials from breeds is constrained to breed region‐specific availability (i.e., whether the breed is native to the region of the specific GB), budget limitations, limited cryogenic tank capacity, maintenance and freezing costs of genetic material, and distances between gene banks and farm zones. The model currently only considers semen collection, accounting for over 90% of materials stored in the GBs covered in this study (Passemard et al., 2018).

### 2.3 Model description

The MIP model is described in terms of sets and indexes, economic parameters, decision variables, objectives functions (OFs) and constraints. Table 1 details model sets, parameters and decision variables. The model is a single‐objective optimization problem, as opposed to multi‐objective formulation; meaning the costs minimization of ((1)) and diversity maximization ((2)) are considered separately. Model equations are as follows.

(1) $$\text{Min Costs}=\sum_{\mathrm{gb}}C_{\mathrm{gb}}$$

**Table 1:** Model description in terms of sets, economic parameters and decision variables

Model inputs	Description	
Sets		
B	Breed	
gb	Gene bank	
Parameters	Description	Value
T	Storage time	1 year
mc_gb_	Gene bank *gb* liquid *N* and storing costs	Table 2
*F*_gb_	Fixed maintenance costs of gene bank *gb*	Table 2
TB	Total conservation budget	600–118,800 EUR
cf_b_	Semen collection cost of gene bank g*b* at farm	Table 3
cc_b_	Semen collection cost of gene bank *gb* at collection point	Table 3
tc_gb_	Travel costs of gene bank *gb*, proportional to distance	2.5 €.km^−1^
*d*_gb_	Distance between gene bank *gb* and farm zone containing breed *b* samples	Table 2
*D*_gb,j_	Distance between gene bank *gb* and gene bank *j*, where *j* is an auxiliary index for gene banks[Link]	Table 4
*A*_b,gb_	Number of doses of breed *b* currently stored in gene bank *gb*	Passemard et al. (2018)
*r*_b_	Number of required samples of breed *b* for collection of semen doses	25 animals
cp_gb_	Capacity of cryotanks in gene bank *gb*	Table 2
μ_b_	Minimum collection in number of doses of semen of breed *b*	0.8 of *M_b_*
*Μ*_b_	Maximum collection in number of doses of semen of breed *b*	cattle (585), sheep(198), goat(105), horse(58), pig(150), poultry(46)
*e*_b,gb_	Binary parameter indicating if breed *b* is available for collection in region of gene bank *gb*	Passemard et al. (2018)
*k*_gb_	Number of doses that gene bank *gb* collects per travel	400 doses
Decision variables		
SB_b,gb_	Amount of doses of semen breed *b* stored in gene bank *gb*	
SF_b,gb,j_	Amount of semen of breed *b* collected by gene bank *gb* at a farm zone in region *j*	
SP_b,gb,j_	Amount of semen of breed *b* collected by gene bank *gb* at a collection point in region *j*	
MC_gb_	Total fixed costs of gene bank *gb*	
VC_gb_	Total variable costs (collection) of gene bank *gb*	
TC_gb_	Variable travels costs (collection) of gene bank *gb*	
CC_gb_	Variable collections costs (collection) of gene bank *gb*	
*C*_gb_	Total costs of gene bank *gb*	

Each GB is associated with its regional vicinity, thus the set of GBs is equivalent to the set of regions. The index *j* is used as an auxiliary to *gb*.

Equation ((1)) represents the least collection costs objective function, given by minimizing the sum of individual gene banks costs, *C_gb_*.

(2) $$\text{Max Diversity}=\sum_{b}\sum_{\mathrm{gb}}SB_{b,gb}$$

Equation ((2)) represents the diversity objective function, defined as the sum over all breeds across the gene banks; where *SB_b,gb_* represents the number of semen doses of breed *b* stored in gene bank *gb*.

(3) $${\text{SB}}_{b,gb}=\sum_{j}\left({\text{SP}}_{b,gb,j}+{\text{SF}}_{b,gb,j}\right)$$

Equation ((2)) defines SB*_b,gb_*in terms of semen *b* collected by gene bank *gb* at a collection point in region *j* (SP*_b,gb,j_*), or semen *b* collected by gene bank *gb* at farm zone *j* (SF*_b,gb,j_*).

(4) $${\mathrm{SB}}_{b,gb}=\left\{\begin{array}{c} \begin{array}{c} 0\\ \mu_{b}\leq SB_{b,gb}\leq M_{b} \end{array} \end{array}\right.$$

Equation ((4)) adds a further constraint to SB*_b,gb_* by imposing the model collects zero, or a value that is in a pre‐defined interval between a minimum number of semen doses of breed *b* (μ*_b_*) and no more than *M_b_* doses.

(5) $$C_{\mathrm{gb}}={\text{MC}}_{\mathrm{gb}}+{\text{VC}}_{\mathrm{gb}}$$

Equation ((5)) defines the total collection and storage costs of a gene bank *gb* as the sum of maintenance costs (MC*_gb_*) and collection costs (VC*_gb_*).

(6) $${\text{MC}}_{\mathrm{gb}}=T*\left(F_{\mathrm{gb}}+{\text{mc}}_{\mathrm{gb}}\sum_{b}{\text{SB}}_{b,gb}\right)$$

Equation ((6)) defines fixed maintenance costs of a gene bank *gb* as the sum of fixed annual costs (*F_gb_*) and variable maintenance costs given by the product of storing and freezing costs of gene bank *gb* (mc*_gb_*) and the total number of semen doses in gene bank *gb* (Σ SB*_b,gb_*).

(7) $${\text{VC}}_{\mathrm{gb}}={\text{CC}}_{\mathrm{gb}}+{\text{TC}}_{\mathrm{gb}}$$

Equation ((7)) defines variable costs as a combination of collection of gene bank *gb* (CC*_gb_*) and travel costs from gene bank *gb* (TC*_gb_*).

(8) $${\text{CC}}_{\mathrm{gb}}=\sum_{b}\left(r_{b}{\text{cf}}_{b}\sum_{j}{\text{SF}}_{b,gb,j}+r_{b}{\text{cc}}_{b}\sum_{j}{\text{SP}}_{b,gb,j}\right)$$

Equation ((8)) represents collection costs in terms of semen doses collected at farm zones, first term in the sum, and at collection points, second term. The first term defines farm collection costs as the total number of breeds *b* collected by gene bank *gb* at all farm zones *j*; sum over *j* (*Σ SF_b,gb,j_*), multiplied by the required number of animal donors per semen dose (*r_b_*) and semen collection costs per dose (*cf_b_*) of breed *b*.

(9) $$\begin{array}{cc} {\text{TC}}_{\mathrm{gb}} & =\frac{1}{k_{\mathrm{gb}}}tc_{\mathrm{gb}}\sum_{b}\sum_{j}\left(d_{j}+D_{gb,j}\right)SF_{b,gb,j}\\ & +\frac{1}{k_{\mathrm{gb}}}tc_{\mathrm{gb}}\sum_{b}\sum_{j}D_{gb,j}SP_{b,gb,j} \end{array}$$

Equation ((9)) describes total travels costs of collections by gene bank *gb* (*TC_gb_*) given by the costs of collections at farm zones and at collection points, respectively the first and second terms in the right hand side (RHS) of Equation ((9)). The first term in the RHS defines the cost of all collections of breeds *b* by gene bank *gb* across all regions *j* (double sum over *b* and *j*). The multiplying parameters inside the sum, *d_j_* and *D_gb,j_* represent the average distance from region of gene bank *j* to farm zones and the distance from gene bank *gb* to gene bank *j*, respectively. The parameter *tc_gb_* represents the average costs per unit of distance; *k_gb_* represents the average number of semen doses collected per journey by gene bank *gb*. The second term in the RHS is analogous to the first term but with *d_j_* =0.

(10) $$\sum_{\mathrm{gb}}\sum_{j}e_{b,j}\left({\text{SF}}_{b,gb,j}+{\text{SP}}_{b,gb,j}\right)=\sum_{\mathrm{gb}}A_{b,gb}$$

Equation ((10)) is used to constrain the model to collect breeds that are currently available in the regional vicinity of the gene banks. The parameter *e_b,j_* is binary vector indicating if breed *b* is available for collection in the region of gene bank *gb* (1 if available, zero otherwise); *A_b,gb_* corresponds to the number of doses of breed *b* currently stored in gene bank *gb*.

(11) $$\sum_{b}{\text{SB}}_{b,gb}\leq {\text{cp}}_{\mathrm{gb}}$$

Equation ((11)) represents gene bank capacities in number of doses of the cryogenic tanks, the total number of doses stored in gene bank *gb* (*∑* SB*_b,gb_*) cannot be greater than the capacity of gene bank *gb*,* cp_gb_*.

(12) $$\sum_{\mathrm{gb}}C_{\mathrm{gb}}\leq \text{TB}$$

Equation ((12)) is the collection budget constraint for the gene banks, where *TB* represents the total European collective budget.

### 2.4 Discontinuous variables

Equation ((4)) introduces a discontinuous variable, SB*_b,gb_*, which increases the model solving complexity and breaks its linearity. The value of SB*_b,gb_* must be either zero or between a particular bound. This is a necessary assumption as the number of stored doses of semen can be zero; but if greater than zero, needs to be between an interval, for example greater than 400 doses but less than 600 doses for cattle breeds. We use a linear programming trick (Bisschop, 2018) to model this discontinuous variable by introducing the indicator variable *y_b_* linked to SB*_b,gb_*:

(13) $$y_{b}=\left\{\begin{array}{c} 0,\;\;for\;\sum_{\mathrm{gb}}{\text{SB}}_{b,gb}\;=0\\ 1,\;\text{for\textbackslash{},}\mu_{b}\leq \sum_{\mathrm{gb}}{\text{SB}}_{b,gb}\leq M_{b} \end{array}\right.$$

The following set of constraints is used to create the desired properties in ((13)):

(14) $$\sum_{\mathrm{gb}}{\text{SB}}_{b,gb}\leq M_{b}y_{b}$$

(15) $$\sum_{\mathrm{gb}}{\text{SB}}_{b,gb}\leq M_{b}y_{b}$$

(16) $$y_{b}\;\text{binary}$$

The model was written in AIMMS algebraic language (Bisschop, 2018), comprising approximately 130,000 variables and 13,000 constraints, written in the matrix form, considering 11 selected European GBs and 489 breeds. It was solved using the CPLEX solver (IBM, 2009).

### 2.5 Data

We obtained model data from two online surveys administered to European institutions holding germplasm and genomic collections as part of the IMAGE project. The first gathered information on species germplasm and breeds. The second focused on cost data, including maintenance, costs related to semen collection and freezing, labour, documentation, average distance between banks to farm zones, costs of skilled labour, materials and equipment and collection failure rates. The information covered six species, namely cattle, sheep, goat, horse, pig and poultry. Eleven banks returned complete information for our analysis (Tables 1 and 2).

**Table 2:** Input data used in the model including the cost parameters, tank capacities and distances

Gene banks	Location	Maintenance cost,F_gb_ (1,000 €.year^−1^)[Link]	Storage cost,mc_gb_ (€.dose^−1^)[Link]	Tanks capacity, *CP*_gb_ (doses)[Link]	Doses currently stored, Σ_b_A_b,gb_ (doses)	Distance to farm zones, d_gb_ (km)[Link]
B1	Paris, France	119	0.12	607,776	303,888	200
B2	Madri, Spain	41	0.56	55,160	27,580	300
B3	Valdepenas, Spain	45	0.30	88,120	44,060	200
B4	Bellaterra, Spain	115	1.89	10,946	5,473	200
B5	Godollo, Hungary	38	3.88	4,124	2,062	200
B6	Thalheim, Gemany	338	0.23	435,174	217,587	100
B7	Wageningen, Netherlands	190	0.05	882,470	441,235	100
B8	P. de Mallorca, Spain	36	0.97	30,018	15,009	100
B9	Kenilworth, UK	115	0.15	551,944	275,972	500
B10	Kiev, Ukraine	115	0.07	292,602	146,301	100
B11	Colmenar V., Spain	115	0.15	335,350	167,675	200

Fixed maintenance costs are composed of labour, property rent and depreciation of tanks and equipment.

Liquid *N* and other storing costs.

Assumed as 50% of current usage.

Round trip distance.

Table 3 describes the collection costs at farm zones comprising management, labour, veterinary and semen freezing costs. Table 4 presents the distances between the GBs considered in this study.

**Table 3:** Collection costs at farm zones and collection points, comprising management, labour, veterinary and semen freezing costs

Collection costs (EUR.animal^−1^)												
	At farm zones, cf_b_						At collection centre, cc_b_					
	Cattle	Sheep	Goat	Horse	Pig	Poultry	Cattle	Sheep	Goat	Horse	Pig	Poultry
B1	100	100	100	150	340	15	182	30	30	50	30	15
B2	50	100	100	150	340	30	50	30	30	50	30	30
B3	100	100	100	150	340	15	182	30	30	50	30	15
B4	100	100	100	150	340	15	182	30	30	50	30	15
B5	100	100	100	150	340	1	182	30	30	50	30	1
B6	100	100	100	150	340	15	182	30	30	50	30	15
B7	300	150	150	300	150	15	300	150	150	300	150	15
B8	60	30	40	60	40	15	60	30	30	60	40	15
B9	500	100	100	150	340	15	500	30	30	50	30	15
B10	30	30	30	30	30	30	30	30	30	30	30	30
B11	100	100	100	150	340	15	182	30	30	50	30	15

**Table 4:** Relative distances between the gene banks (in km)

Distance between gene banks, D_gb,j_ (km)											
	B1	B2	B3	B4	B5	B6	B7	B8	B9	B10	B11
B1	0	1,274	1,478	1,028	1,538	1,045	495	1,290	640	2,402	1,238
B2	1,274	0	214	611	2,572	2,213	1,761	740	1,900	3,687	39.7
B3	1,478	214	0	682	2,644	2,285	1,963	679	2,103	3,789	250
B4	1,028	611	682	0	1,962	1,602	1,498	290	1,658	3,107	631
B5	1,538	2,572	2,644	1,962	0	501	1,374	2,247	1,950	1,094	2,593
B6	1,045	2,213	2,285	1,602	501	0	1,027	2,034	1,603	1,362	2,379
B7	495	1,761	1,963	1,498	1,374	1,027	0	1,780	697	1,912	1,731
B8	1,290	740	679	290	2,247	2,034	1,780	0	1,920	3,369	728
B9	640	1,900	2,103	1,658	1,950	1,603	697	1,920	0	2,578	1,870
B10	2,402	3,687	3,789	3,107	1,094	1,362	1,912	3,369	2,578	0	3,657
B11	1,238	39.7	250	631	2,593	2,379	1,731	728	1,870	3,657	0

### 2.6 Scenarios and sensitivity analysis

The baseline scenario *S_0_* represents the current configuration of breed collections distributed across the 11 GBs. The analysis assumes that breeds currently available in each bank are native to the respective region; that is, if a given cattle breed *b1* is stored in BANK 1 in *S_0_*, it can only be collected from region 1. The optimized scenario *S_UC_* represents the minimum cost breed‐gene bank allocation allowing cross‐region collection, and assumes hypothetically an unlimited capacity of cryogenic tanks. *S_C50_* is analogous to *S_UC_* but assumes that current bank breeds occupy 50% of tank capacities. *S_1_*,* S_2_*,..., *S*_11_ assume unlimited capacities and impose one bank to store all breeds. That is, *S_1_* means all breeds across the 11 regions are collected by BANK 1 only, and analogously for *S_2_* to *S_11_*.

While the previous scenarios are explored by minimizing collection costs, a sensitivity analysis employs a diversity function (Equation (2)) to estimate the cost per conserved breed for cattle, sheep, goat, poultry and pigs, depending on available budget. We define a diversity value (*D_i_*), measured by the number of selected breeds, as a function of *n* available budgets (*B_i_*) as follows:

(17) $$B_{i}=B_{0}+i\frac{\left(B_{\text{Max}}-B_{0}\right)}{n}$$

where *B_0_* represents the initial budget; *i* represents the budget scenario and varies from 1 to *n* = 100 (an arbitrary number of scenarios); and *B*_Max_ is the maximum available budget. For all *B_i_*'s, the value of diversity is calculated as *D_i_* = f(*B_i_*). Where *f*(*B_i_*) represents the optimal solution of the MIP model when maximizing the diversity function. *D_i_* and *B_i_* are plotted as y‐and x‐axis to define efficient cost curves for each of the livestock species.

## 3 RESULTS

**Figure 2:**
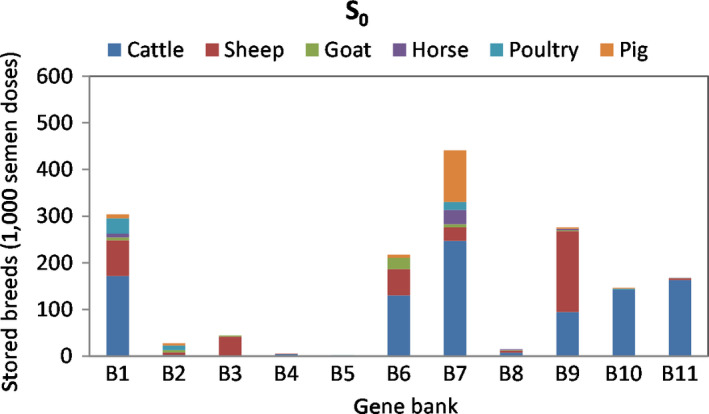
Baseline (*S*_0_) gene bank allocation (total number of doses within a species) across the 11 European banks for livestock breeds [Colour figure can be viewed at wileyonlinelibrary.com]

The estimated total cost of the current breed allocations for the 11 European GBs is 23.2 M EUR, including regional collection costs only because the S_0_ scenario assumes no cross‐regional collection strategies. Of the 489 breeds, 55% of semen doses are cattle, 25% are sheep, 9% pig, 4% poultry, followed by goat and horse, together representing 3% of the total collected doses. Figure 2 shows how breeds, according to species, are distributed across the GBs. Most cattle breeds are distributed between B1, B6, B7, and B9 to B11. While 78% of sheep breeds are in B1, B6 and B9. Pig breeds are mostly in B7 (82%). Analysis of *S_0_* shows overlapping collection of cattle, goat, sheep and pigs, and most significantly for cattle; for example, the cattle breed Blonde D'Aquitaine is currently stored in five different banks, varying from 50 semen doses (B11) to 9,670 (B1) (see Appendix Table A1 for details).

**Figure 3:**
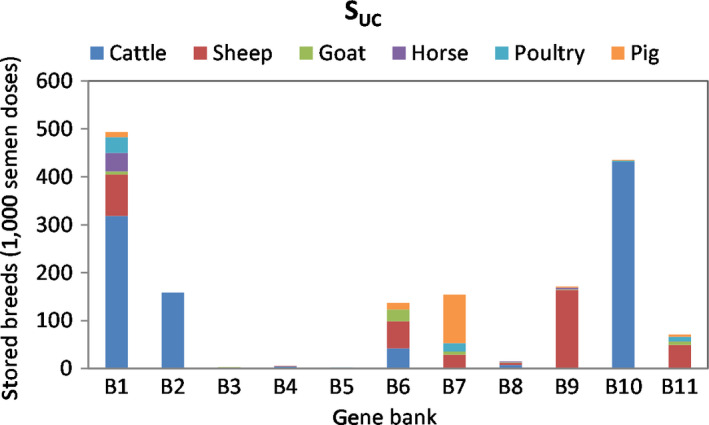
Least cost gene bank allocation (in total number of doses within a species) across the selected 11 European banks for livestock breeds under unlimited capacity of cryogenic tanks, scenario *S*_UC_ [Colour figure can be viewed at wileyonlinelibrary.com]

Figure 3 shows the alternative least cost collection strategy (*S_UC_*). If the breeds in Figure 2 were collected at least cost, the required budget would fall by 23%, or around 5.4 M EUR. Figure 3 shows *S_UC_* reduces collection costs by transferring cattle breeds from GBs 6, 7, 9 and 11 to GBs 1, 2 and 10, relative to *S_0_*, sheep breeds from GB 3 to GB 11, horse breeds from B7 to B1, while poultry and pigs are kept approximately the same as S_0_. In fact, Table 2 shows those cryogenic banks have relatively low fixed and collection costs, and are geographically closer to other collection regions (as shown in Table 4).

**Figure 4:**
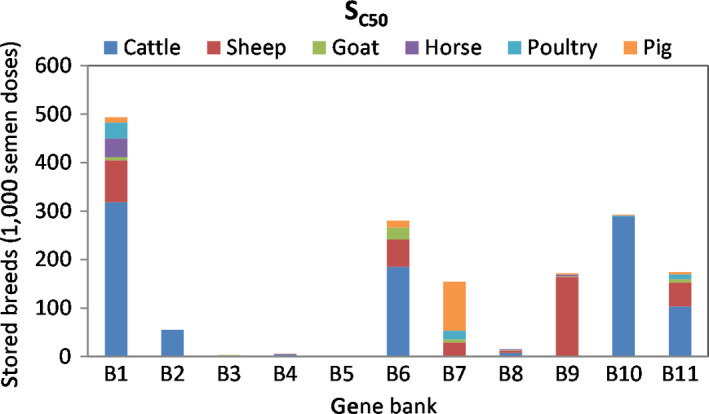
Least cost gene bank allocation (in total number of doses within a species) across the selected 11 European banks for livestock breeds under limited capacity of cryogenic tanks, scenario *S*_C50_ [Colour figure can be viewed at wileyonlinelibrary.com]

Figure 4 shows that assuming the GBs are currently operating at 50% of their capacities (*S_C50_*) reduces the required budget by 19% relative to *S_0_*, or by 4.3 M EUR. Figure 4 shows that the strategy for minimizing costs under limited capacity, in relation to S_UC_, is transferring cattle breed doses from B2 and B10 to B6 and B11 due to the first two being above the gene banks capacity. This is because under *S_UC_* only BANK 2 exceeds the assumed tank capacity, thus moving to the closest GB, BANK 11, which is only 39.7 km away (Table 4) minimizes collection costs.

**Figure 5:**
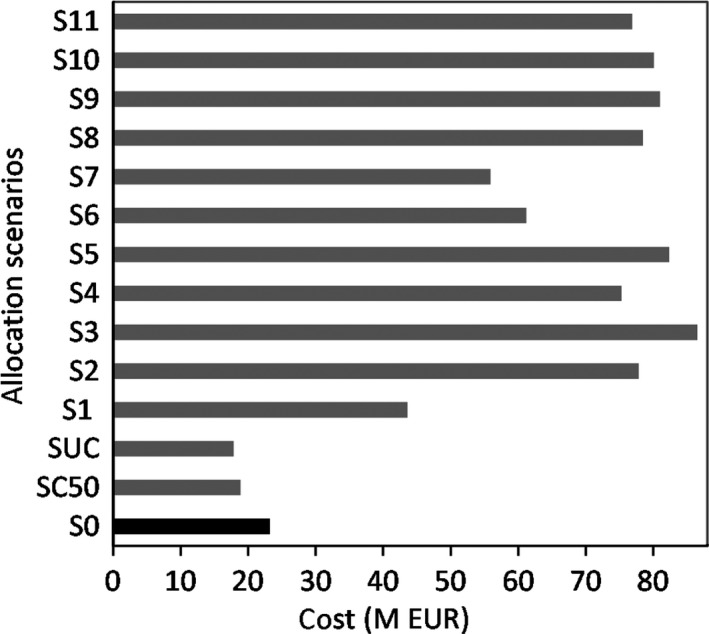
Gene bank allocation scenarios and associated costs. S_0_, S_C50_ and S_UC_ are the current configuration of breed collections, constraint and unconstraint minimum cost collection scenarios, respectively. Scenarios S1 to S11 represent single gene bank allocations; S1 means all breeds are collected by BANK 1 only, and analogously from S2 to S11

The alternative single gene bank scenarios *S*_1_ to *S*_11_ are presented in Figure 5, showing costs varying by +100% to 285% relative to *S*_0_. The difference is explained by an inefficient collection strategy that ignores the relative breed costs across different GBs and the variation in travel costs, which are in turn related to the number of doses collected by a bank per visit to a region and farm zone.

**Figure 6:**
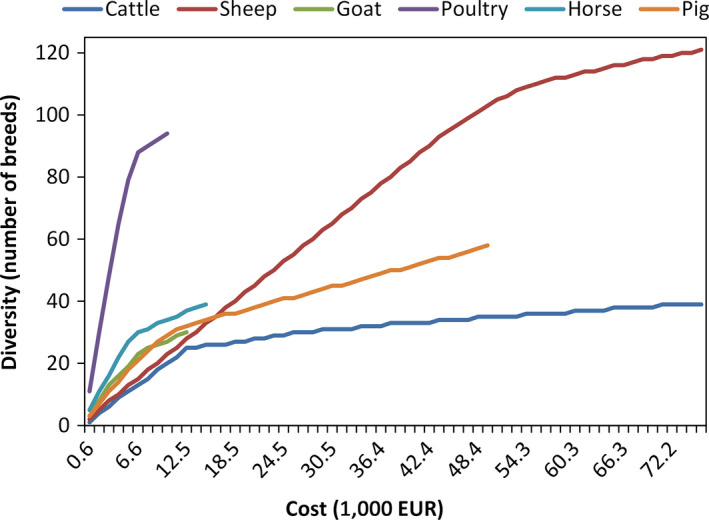
Sensitivity analysis of diversity as a function of collective EU budget for livestock breeds [Colour figure can be viewed at wileyonlinelibrary.com]

Figure 6 shows diversity cost curves for cattle, sheep, goat, poultry, pigs and horse. Increasing marginal cost per stored breed reflects the fact that breed collection takes place in the same region as the banks (i.e., native breeds). As the available budget (or cost) increases (x‐axis), more genetic material from more breeds can be collected, although at higher cost and requiring more cross‐regional collection due to cryogenic tank capacity limitations (upper cost) up to the point where all available breeds within the GBs are collected, as the model assumes unlimited tank capacity for constructing the diversity cost curves. Collection and storage cost per breed varies between 55 and 2,531 EUR, depending on the number of breeds and species that are already collected.

## 4 DISCUSSION

Model results suggest a potential for cost saving across European cryogenic banks by strategic collection and conservation planning. The results indicate overlapping collections across the GBs. However, from a genetic viewpoint the same breed in different countries may harbour different genetic diversities. Despite we do not consider transboundary breeds those will not change the results as they represent a small fraction of the total breeds considered in this study (21 out of 489).

As well as being risky, collecting all materials in one of the existing cryogenic banks considered in this study is a relatively expensive option compared with multiple banks. This is because there is no GB with storage and collection costs cheaper for all species, and storing in a single GB increases cross‐country transportation costs because some breeds are unavailable for collection in a single region.

Our results could be refined by improved cost estimates for collections and the addition of more breeds and country collections not taken into account in the analysis, for example, from FAO DAD‐IS database (FAO‐DADIS, 2018). However, adding these will also require collection of further regional cost data, which are usually incomplete. For example the cost surveys used in this study (Passemard et al., 2018) revealed inconsistent approaches to the recording of collection and storage costs, and different ways of recording units of collected materials.

A further apparent limitation is the representativeness of the cost survey, or more specifically varying banking technologies. A bank with lower storage costs may imply poorer conservation quality and higher failure risks, which we would ideally include as a specific variable in the optimization problem.

The analysis further suggests that the diversity‐cost curve varies for breeds of each species. Since the surveys did not seek to understand breed‐specific weighting criteria, the diversity‐cost curves do not discriminate in terms of important breed‐specific attributes, for example, related to greater or lower expected economic returns, breed endangerment, susceptibility to climate change or cultural attributes.

Like relative failure risks, these attributes could be included in the MIP as stochastic parameters, for example by forcing the model to select breeds with probability of extinction greater than a threshold value, variance of expected return, or probability of successful restoration. Assignment of breed‐specific and technology attribute weights to the diversity function will change the shape of the curves.

Many breed attributes are likely to be prominent in any survey of public preferences for conservation spending, which we suspect would mirror priorities for public good provision related to in situ conservation decisions. However, this raises a further question about the demand for ex situ collections, and whether they should serve public or private good objectives; the latter focussed largely on animal productivity traits. This largely depends on ownership and how they are financed. The survey by Passmard et al (2018) suggested that most of the respondent collections were under the auspices of public institutions. However, the same survey did not seek views on how this translates into objectives for attribute selection. Furthermore, the survey most likely overlooks other genetic material held in private (i.e., industrial) collections. Accordingly, without clarity on private sector preferences we can only optimise over a known proportion of the stored resources.

## 5 CONCLUSION

Rapid progress in the development of next generation gene sequencing and bioinformatic tools have revolutionised animal breeding, but potentially distracted from a basic problem of what genetic and reproductive materials to collect and store, and how stored information is consistently recorded. Breed and gene bank selection clearly involves numerous biotechnological, institutional and economic challenges that can be informed by mathematical modelling of cost‐effective breed conservation.

For given objectives and constraints our model provides some indication of potential rationalization options and demonstrates the increasing marginal costs of conservation effort. The exercise begs important questions about the specific optimization objectives, which in turn require more institutional coordination to define the mix of private and public good objectives and hence potential cost and benefit sharing. This implies clearer articulation of in situ risks including endangerment due to climate change and other pressures, expected economic returns and other attributes that determine stakeholders’ conservation preferences. There is also a need to improve understanding of the efficacy of technologies developed for ex situ curation and associated risks of successful use in future agricultural scenarios.

## CONFLICT OF INTEREST

The authors declare no conflict of interest.

## APPENDIX 1

**Table A1:** Overlapping breeds and number of semen doses in the GBs

Breed	Number of semen doses								Total
	B1	B2	B3	B6	B7	B9	B10	B11	
Cattle ‐ Belgian Blue				1,150	375				1,525
Cattle ‐ Blonde D´aquitaine	9,670			350	75		770	50	10,915
Cattle ‐ Brown Swiss				15,344	87				15,431
Cattle ‐ Charolaise	11,600			672			1,649	4,396	18,317
Cattle ‐ Galloway				100		711			811
Cattle ‐ Hereford						486	2,000		2,486
Cattle ‐ Holstein					142,944		36,040		65,547
Cattle ‐ Jersey					100		1,050		1,150
Cattle ‐ Limousine	7,000			1,650			3,539	2,447	14,636
Cattle ‐ Montbeliard	21,100			92	75		218		21,485
Cattle ‐ Piedmont				100	25		3,000		3,125
Cattle ‐ Simmental				86,200	25		16,914		103,139
Goat ‐ Murciano Granadina			1,337					43	1,380
Goat ‐ Saanen	923				75				998
Pig ‐ Duroc	287				2,378				2,665
Pig ‐ Landrace	298			200					498
Pig ‐ Large White				134		250			384
Pig ‐ Pietrain				602	7,033				7,635
Sheep ‐ Manchega		725	39,794					3,043	43,562
Sheep ‐ Romaney	2,534					2,402			4,936
Sheep ‐ Suffolk	5,509					7,434			12,943
